# Harnessing cellular aging in human stem cell models of amyotrophic lateral sclerosis

**DOI:** 10.1111/acel.12862

**Published:** 2018-12-19

**Authors:** Oliver J. Ziff, Rickie Patani

**Affiliations:** ^1^ The Institute of Neurology University College London London UK; ^2^ The Francis Crick Institute London UK

**Keywords:** aging, amyotrophic lateral sclerosis, motor neuron disease, neurodegeneration, pluripotent stem cells

## Abstract

Amyotrophic lateral sclerosis (ALS) is a relentlessly progressive neurodegenerative condition that is invariably fatal, usually within 3 to 5 years of diagnosis. The etiology of ALS remains unresolved and no effective treatments exist. There is therefore a desperate and unmet need for discovery of disease mechanisms to guide novel therapeutic strategies. The single major risk factor for ALS is aging, yet the molecular consequences of cell type‐specific aging remain understudied in this context. Induced pluripotent stem cells (iPSCs) have transformed the standard approach of examining human disease, generating unlimited numbers of disease‐relevant cells from patients, enabling analysis of disease mechanisms and drug screening. However, reprogramming patient cells to iPSCs reverses key hallmarks of cellular age. Therefore, although iPSC models recapitulate some disease hallmarks, a crucial challenge is to address the disparity between the advanced age of onset of neurodegenerative diseases and the fetal‐equivalent maturational state of iPSC‐derivatives. Increasing recognition of cell type‐specific aging paradigms underscores the importance of heterogeneity in ultimately tipping the balance from a state of compensated dysfunction (clinically pre‐symptomatic) to decompensation and progression (irreversible loss of neurological functions). In order to realize the true promise of iPSC technology in ALS, efforts need to prioritize faithfully recapitulating the clinical pathophysiological state, with proportionate emphasis on capturing the molecular sequelae of both cellular age and non‐cell‐autonomous disease mechanisms within this context.

**Abbreviations**
ALS: amyotrophic lateral sclerosis
ARTAG: aging‐related tau astrogliopathy
CNS: central nervous system
dMMP: matrix metalloproteinase 1
ESC: embryonic stem cells
GFAP: glial fibrillary acidic protein
iPSC: induced pluripotent stem cells
LMNA: lamin A
MND: motor neuron disease
NMJ: neuromuscular junction
SOD1: superoxide dismutase 1

## 1 INTRODUCTION

As life expectancy of the population increases, the prevalence of aging‐associated disorders, such as amyotrophic lateral sclerosis (ALS) and other forms of neurodegeneration, is also increasing (Lutz, Sanderson, & Scherbov, 2008). Regrettably, in contrast to the escalating affliction that these neurological disorders have on society, effective therapeutics are far from realization. Overall, animal models have provided crucial insight across the range of basic and applied neuroscience. However, despite hundreds of clinical trials based on pre‐clinical studies using animal models, not one has yielded a significant therapy that is of real clinical impact for ALS. This may reflect crucial interspecies differences, which are increasingly documented across molecular, cellular, circuit‐level, functional, and anatomical domains. Human postmortem tissue overcomes this problem to some degree, but it also represents an advanced stage of the disease process, making it impossible to capture initiating molecular pathogenic events.

Importantly, these limitations can now be overcome, at least in part, by using induced pluripotent stem cells (iPSCs). Differentiating pluripotent stem cells into enriched cell type‐specific populations presents an exciting opportunity to attain large numbers of patient‐specific cells in vitro, to model disease, and test drug candidates. The field has seen a rapid advancement in the establishment of iPSC technology with key molecular pathogenic signatures detected across a wide spectrum of disease models (Dimos et al., 2008; Hall et al., 2017; Park et al., 2008). Despite this progress, current differentiation protocols produce cells that possess fetal‐equivalent maturation, which contrasts greatly with age‐related pathologies like late‐onset neurodegenerative diseases (Arbab, Baars, & Geijsen, 2014; Liu, Ding, & Izpisua Belmonte, 2012; Luisier et al., 2018; Mertens et al., 2015; Miller et al., 2013; Patani et al., 2012; Vera, Bosco, & Studer, 2016). Whilst relevant phenotypes have successfully been reported in iPSC models, they resemble early stage pathogenic events, rather than age‐related degenerative features of the condition. Consequently, to accurately elucidate age‐related phenotypes, the erasure of the hallmarks of biological age caused by reprogramming should arguably be reinstated. Strategies to circumvent this hurdle involve inducing cellular age using a variety of approaches discussed herein. Indeed proof of principle studies have shown that this approach enables iPSC models to capture some authentic aging‐related phenotypes (Mertens et al., 2015; Miller et al., 2013; Paavilainen et al., 2018; Vera et al., 2016). We review strategies that have been developed to induce cellular age in order to overcome the rejuvenating effect of reprogramming. These approaches should further advance the molecular, cellular, and functional production of patient‐derived iPSC platforms to investigate ALS with fidelity and precision.

## 2 INDUCING PLURIPOTENCY REVERSES KEY HALLMARKS OF CELLULAR AGE

**Figure 1:**
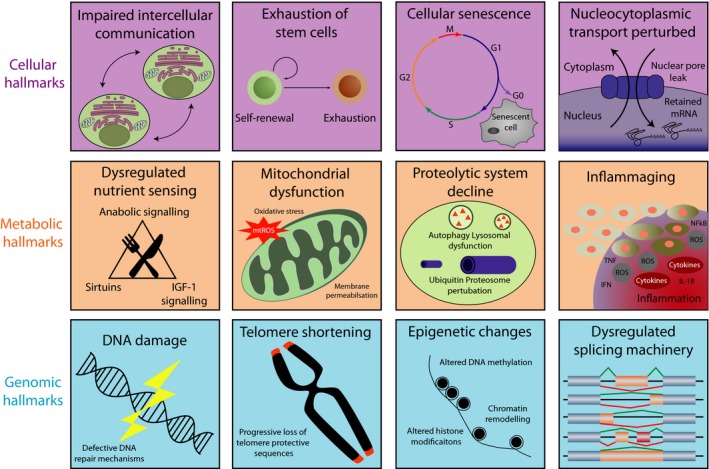
Hallmarks of aging. Hallmarks of aging as previously described by Lopez‐Otin et al. (2013). We add perturbation of nucleocytoplasmic transport; inflammaging; and splicing changes whilst segregating hallmarks into cellular, metabolic, and genomic categories

Aging is a highly pleiotropic process making it difficult to discern between the accumulation of damage throughout life or conversely an active program. Cumulatively, aging precipitates alterations that damage both structural and functional aspects of cellular physiology, which are summarized in Figure 1 and have been extensively reviewed elsewhere (Lopez‐Otin, Blasco, Partridge, Serrano, & Kroemer, 2013). Reprogramming of somatic cells back to the pluripotent state effectively *deletes *molecular traces of aging and maturation that were acquired during the life of the somatic cell, resetting the biological clock so that adult cells become tantamount to embryonic stem cells (ESCs) (Miller et al., 2013; Studer, Vera, & Cornacchia, 2015). Reprogramming also has the capability of reversing aging‐related epigenomic alterations, such as DNA methylation marks that human tissues acquire over time. Indeed the restoration of DNA methylation during in vitro differentiation can serve as a marker of iPSC maturity (Horvath, 2013).

**Figure 2:**
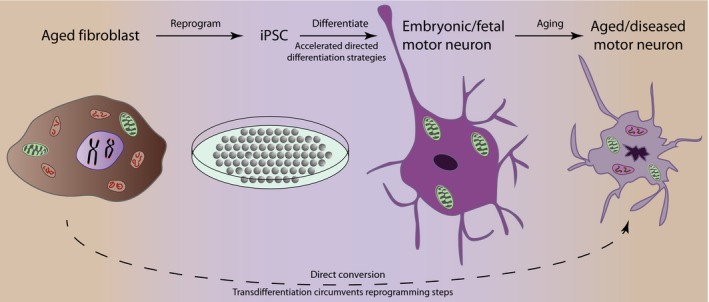
Process of cellular reprogramming and directed differentiation. Patient‐derived somatic cells are cultured and reprogrammed to a pluripotent state by expression of pluripotency factors. Patient‐specific iPSCs are differentiated toward motor neurons following neural induction and patterning. These motor neurons remain embryonic‐like, unless a further aging phase is induced. Induced ageing of iPSC models or transdifferentiation offer routes to bypass the reprogramming and differentiation steps thus preserving cellular age. iPSCs: induced pluripotent stem cells

Induced pluripotent stem cells have been successfully differentiated into myriad cell types relevant to ALS (reviewed in Patani, 2016; Tyzack, Lakatos, & Patani, 2016). These directed differentiation protocols generate cell types that represent fetal, rather than adult, maturity. We and others assessed this issue by comparing genome‐wide expression profiles of human pluripotent stem cells, multipotent neural precursor cells, and terminally differentiated neurons with fetal and adult postmortem counterparts (Mertens et al., 2015; Miller et al., 2013; Patani et al., 2012). This was supported by a more recent global gene expression and network analysis, investigating iPSC‐derived motor neurons, which confirmed that these were fetal in maturational state based on their transcriptomic profiles (Ho et al., 2016; Luisier et al., 2018) (Figure 2).

## 3 **DIRECTED DIFFERENTIATION OF **iPSCs** PERMITS CAPTURE OF EARLY DISEASE PHENOTYPES**

The first iPSC model of human neurological disease studied spinal muscular atrophy (SMA) type 1, which selectively affects lower motor neurons and presents early in the postnatal period. This study successfully recapitulated motor neuron degeneration and death. However, even in this early‐onset disease there are key postnatal motor neuron defects that appear in the absence of functioning SMN (survival motor neuron) protein, such as axon truncation, loss of NMJs, and reduction in branching and outgrowth (Ebert et al., 2009). Accordingly, iPSC‐derived motor neurons from SMA type I patients developed more widespread disease hallmarks only after extended in vitro culture for several months (Corti et al., 2012; Ebert et al., 2009).

Conventional (“fetal‐like”) iPSC‐derived motor neurons and astrocytes have also demonstrated clear value in capturing early biochemical phenotypes of ALS in a cell type‐specific fashion (Hall et al., 2017; Luisier et al., 2018; Simone et al., 2018; Tyzack et al., 2017). Although the iPSC technology allows capture of the earliest molecular pathogenic events, elucidating the contribution of cell type‐specific aging programs is a key consideration for future disease modeling. Therefore, despite considerable progress, cellular rejuvenation resulting from reprogramming to the pluripotent state raises concern over missing salient age‐related cellular phenotypes (Hu et al., 2010; Patterson et al., 2012). To model ALS with fidelity, inducing a cellular age that resembles late adulthood is desirable in order to discriminate age‐related cellular phenotypes (Figure 2). Despite challenges in inducing cellular age of iPSC‐derivatives, various approaches have been proposed for manipulating the aging process, largely inspired by important work in animal models.

### 3.1 *Inducing cellular aging—lessons from *in vivo* studies*

Experimental models, including heterochronic (differentially aged) parabiosis, where the circulatory systems of young and old mice are surgically connected, have identified systemic factors that influence aging (Conboy, Conboy, & Rando, 2013). These experiments demonstrated that not only does blood from young mice rejuvenate older mice, but young mice undergo accelerated aging when subjected to blood from older mice (Villeda et al., 2014). Although the identity of all specific aging factors in human plasma remains incomplete, significant insights into the molecular underpinnings of this process have recently been achieved. Human umbilical cord plasma was shown to revitalize the hippocampus, and tissue inhibitor of metalloproteinases 2 (TIMP2) was identified as a plasma protein that increases synaptic plasticity and cognitive function in aged mice (Castellano et al., 2017). In vivo studies, using both systemic administration and inhibition of plasma candidates found in young blood, have revealed other factors that slow age‐dependent neural deterioration and induce reversing of aging, including (a) growth differentiation factor 11 (Katsimpardi et al., 2014); (b) oxytocin (Elabd et al., 2014); and (c) Delta–Notch signaling (Honoki, 2017). Conversely, using a mouse model to study muscle stem cells, activation of the canonical Wnt signaling pathway promoted conversion from a myogenic to an aging‐associated fibrogenic lineage, thereby implicating a possible bidirectional instructive regulation (Brack et al., 2007).

Key drivers of these aforementioned regulators of aging have also been studied. Recognition that mouse aging involves the loss of hypothalamic stem cells inspired ablation studies which consistently led to accelerated aging and lifespan shortening (Zhang et al., 2017, 2013). Conversely, aging delay and lifespan extension were attained in middle‐aged mice when implanted with healthy hypothalamic stem cells. This recovery of physiological processes was shown to be mediated, at least in part, through release of exosomal miRNAs into the cerebrospinal fluid (Zhang et al., 2017). Whether this in turn leads to a series of downstream signaling events in a theoretical aging‐cascade remains to be studied. Given the myriad of aging regulators that have been discovered, it is likely that there are multiple sequential steps, with various molecular sub‐regulators at different end‐organ sites that ultimately influence the overall net rate of aging in a given tissue.

## 4 INDUCING CELLULAR AGING IN IPSC‐DERIVATIVES

An important challenge in the iPSC field is to specifically modulate the biological age of cultures independently of their chronological age. As the sequence of iPSC differentiation in vitro reflects lineage determination during embryogenesis, the reason a prolonged culture enables maturation is likely due to the presence of a built‐in clock that guides the tempo and direction of cell fate. This cell‐autonomous “pacemaker” phenomenon is supported by the finding that in vitro neurogenesis is temporally cataloged into distinct phases, and mirrors in vivo neural development (Hu et al., 2010). These findings, which are species‐specific, suggest that an internal timer regulates cellular maturation and aging. This raises the prospect of predictably manipulating master regulators of this mechanism experimentally, which may in turn permit the accurate delineation of age‐related phenotypes (Shi, Kirwan, Smith, Robinson, & Livesey, 2012).

**Figure 3:**
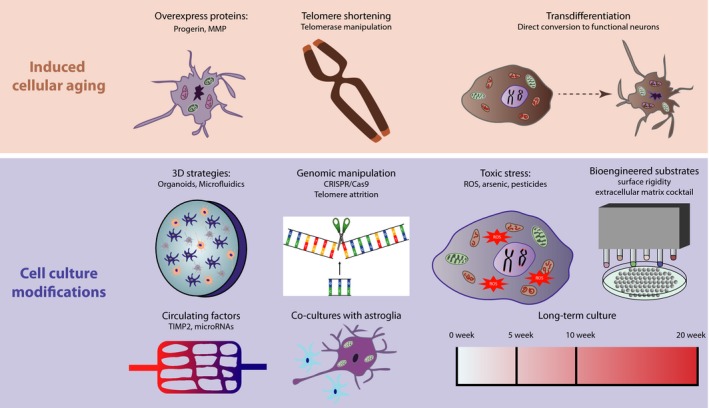
Strategies to model cellular aging. Throughout maturation and aging somatic cells undergo functional and gene expression alterations, which are considered to be crucial in the onset of neurodegenerative conditions such as ALS. The iPSC field has previously been constrained by retained fetal‐like phenotypes within cultured motor neurons. Using induced cellular aging strategies and cell culture modification approaches to model aging has significantly improved these approaches

Here, we discuss the approaches that have been attempted to induce cellular aging of iPSC‐derived neurons. Importantly, we distinguish the approaches that truly attempt to model cellular aging (discussed below; see Table 1), from those that merely expedite routes of generating fetal‐equivalent neurons (Figure 3; and Supporting Information Box S1).

**Table 1:** Strategies to induce cellular aging

Method	Detail	Strengths	Limitations	References
Progerin overexpression	Progerin overexpression to induce aging in an induced pluripotent stem cell (iPSC) model of neurodegeneration	Marked dendrite degeneration, loss of neuronal subtype‐specific expression, enlarged mitochondria, disease‐specific inclusions	Does not capture all aspects of aging	Miller et al. (2013) Zhang et al. (2017) Liu et al. (2011) Nissan et al. (2012) Scaffidi and Misteli (2008)
Telomere shortening	Pharmacologically reduced telomerase activity with a small molecule inhibitor in iPSC‐derived neurons	DNA damage, mitochondrial ROS generation, and dendrite degeneration	Telomere length remained variable	Vera et al. (2016) Harel et al. (2015) Marion et al. (2009)
Transdifferentiation	Direct conversion of patient‐derived somatic cells into mature‐specific cell type of interest. Reprogramming is circumvented, and cellular age is preserved.	Retain age‐associated transcription traits and functional deficits of the donor cell population. Accumulation of mutant protein aggregates, DNA damage, heterochromatic loss, cellular senescence, and mitochondrial dysfunction	Limited cellular supply Reliant on knowing which factors to express	Mertens et al. (2015) Victor et al. (2018) Son et al. (2011) Park et al. (2012) Tang et al. (2017) Vierbuchen et al. (2010)

### 4.1 Progerin expression

Although the systemic factors that induce aging are still mostly unidentified, some genetic mutations have been found that accelerate aging. Hutchinson–Gilford progeria syndrome (HGPS), caused by a point mutation in the lamin A (LMNA) gene, is one such example of a disease characterized by accelerated aging (Scaffidi & Misteli, 2008). Alternative post‐transcriptional modification of the LMNA mRNA transcript results in translation into a truncated farnesylated form of lamin A protein, termed progerin. Lamins are vital constituents of the nuclear envelope and are integral to most aspects of nuclear metabolism. Progerin itself acts to damage and shorten telomeres, and activates DNA damage checkpoints, inhibiting cell proliferation and encouraging senescence. Modeling HGPS in iPSC‐derived neurons revealed cell type‐specific protection from the aging effects of progerin as neurons are deficient in lamin A protein (Liu et al., 2011), as a result of expression of microRNA 9 (Nissan et al., 2012).

Although progerin overexpression has not yet been utilized in an iPSC model of ALS, it has been applied to iPSC‐derived midbrain dopaminergic neurons in a Parkinson's disease (PD) model (Miller et al., 2013). This approach successfully generated an aged phenotype, as demonstrated by neuromelanin accumulation, marked dendrite degeneration, loss of tyrosine hydroxylase expression, and enlarged mitochondria or Lewy body‐precursor inclusions, producing a more representative PD model. Although this indicates that progerin exposure is sufficient to induce some aging‐associated markers, questions remain as to whether this approach faithfully captures key aspects of authentic neuronal aging.

### 4.2 Telomerase inhibition

Another approach to induce aging involves altering one of the classic hallmarks of aging, telomere shortening. With age, the telomere preserving enzyme, telomerase is down regulated and telomeres get successively shorter with each cell cycle. Vera and coworkers utilized a pharmacological approach to reduce telomerase activity with a small molecule inhibitor, thereby shortening telomeres of iPSC‐derivatives. The iPSC‐derived neurons with shorter telomeres exhibited aging‐related features, including DNA damage, mitochondrial ROS generation, and dendritic atrophy (Vera et al., 2016). Although this approach successfully produced some disease‐related features, in a model of PD telomere length remained variable and the full impact of telomere shortening on post mitotic cells has yet to be systematically resolved.

### 4.3 Transdifferentiation

An alternative approach to bypass the issue of the embryonic nature of iPSCs is direct conversion of patient‐derived somatic cells into the mature‐specific cell type of interest. In this way, reprogramming to the embryonic state is circumvented and cellular age is preserved. Mertens and coworkers directly converted aged donor fibroblasts into neurons by overexpressing the proneural genes Ascl1 and Ngn2 combined with a cocktail of small molecules to enhance direct conversion efficiency. Using transcriptional profiling, they demonstrated that these cells retained their age‐associated transcription traits and functional deficits of the donor cell population (Mertens et al., 2015). This study was recently independently confirmed by Victor et al. (2018), who used microRNA‐based direct neuronal conversion of patient fibroblasts into neurons. The preservation of donor cellular age here allowed the identification of phenotypes that were not evident using the conventional iPSC‐based directed differentiation. This underscores the value of retaining donor cellular age status when modeling late‐onset neurological disorders, and by comparison to their iPSC‐derived counterparts, it is possible to discriminate age‐related cell type‐specific phenotypes.

Such strategies are clearly applicable to iPSCs from ALS patients. Indeed, overexpression of key transcription factors involved in motor neuron development (Olig2, Hb9, Asc1, Lhx3) transforms somatic cells directly into induced motor neurons (Park et al., 2012; Son et al., 2011). By applying these transcription factors to both primary fibroblasts and iPSC‐derived cells, key aging hallmarks (DNA damage, heterochromatin loss, and increased cellular senescence) were only preserved with direct conversion to motor neurons and not with induced pluripotency. As might be predicted, the iPSC‐derived motor neurons were rejuvenated and failed to retain the crucial aging hallmarks from their donors (Tang, Liu, Zang, & Zhang, 2017). This comparative analysis of transdifferentiation vs. reprogramming demonstrates that transdifferentiation holds significant value for modeling age‐related aspects of ALS pathobiology (Table 2).

**Table 2:** Comparison of in vitro paradigms to model human neurodegeneration

Aging Hallmark	induced pluripotent stem cells (iPSC)‐derived neurons	Aged iPSC‐derived neurons
Perturbed cellular architecture and functional maturity	Intact (fetal‐equivalent) cellular architecture which do not fully capture age‐related cellular phenotypes. Dendrite length and number are not significantly affected. Cells are less prone to activating cell death program. Electrophysiological immaturity with resting membrane potential relatively depolarized, input resistance raised, with slower kinetics and smaller amplitude.	Aged cells have enhanced nuclear folding and blebbing. The nuclei are disorganized and appear misshapen. Reduced number and shorter dendrites. Cells exhibit increased susceptibility to apoptosis and cellular stressors. Electrophysiological maturity with respect to resting membrane potential, capacitance, action potential threshold, and amplitude.
Impaired nuclear‐cytoplasmic compartmentalization	iPSC exhibit tightly regulated compartmentalization of nuclear and cytoplasmic proteins as well as the nuclear pore complexes.	Age‐dependent loss of nucleocytoplasmic compartmentalization. Cells display age‐associated decreases in nuclear transport with loss of nuclear cytoplasmic receptors for example, RanBP17. Higher levels of soluble detergent‐resistant TDP‐43.
Gene expression signature and splicing	The transcriptome exhibits no molecular features of aging	Display an age‐specific transcriptional profile. Return of differentially expressed gene transcripts found in aged neurons involved with aging, synaptic transmission, neuron generation and differentiation, stress response, inflammation, calcium homeostasis, nuclear pore, and splicing.
Epigenetic	Methylation signatures resemble embryonic stem cells (ESCs). However, after reprogramming iPSCs harbor residual DNA methylation signatures from their donor cells (epigenetic memory) with preference for differentiation into their original cell lineage	Epigenome modifications and histone methylation patterns are similar to aged neurons with net loss of heterochromatin, although there are reproducible increases in DNA methylation at some CpG sites and decreases at others. These alterations are detectable prior to aberrations in cellular architecture.
Telomeres	Telomere length resembles ESCs; however, heterogeneity in telomere length has been found.	Length of telomeres is abrogated, and extended culture leads to progressive telomere shortening and loss of self‐renewal.
DNA damage	DNA repair mechanisms function efficiently.	There is accumulation of DNA damage (gamma H2AX) and reduced capacity of the DNA repair mechanisms leading to senescence reflecting cellular aging.
Mitochondrial dysfunction	Glycolysis > mitochondrial oxidative metabolism, resembling ESCs.	Aged neurons display decreased oxidative phosphorylation‐related gene expression, impaired axonal mitochondrial morphologies, lower mitochondrial membrane potentials, reduced energy production, and increased mitochondrial radical oxygen species which leads to increased oxidized proteins.
References	Cooper et al. (2012), de Boni et al. (2018), Israel et al. (2012), Lister et al. (2011), Mertens et al. (2015), Miller et al. (2013), Nguyen et al. (2011), Ohi et al. (2011), Seibler et al. (2011), Simara et al. (2017), Wang et al. (2012), Xu et al. (2013)	Batista et al. (2011), Gunhanlar et al. (2018), Ho et al. (2011), Kim et al. (2018), Liu et al. (2011), Mertens et al. (2015), Miller et al. (2013), Nguyen et al. (2011), Simara et al. (2017), Vera et al. (2016), Zhang et al. (2011)

## 5 DO THESE STRATEGIES CAPTURE AUTHENTIC CELL TYPE‐SPECIFIC SIGNATURES OF AGING?

Although the aforementioned proof of concept studies recapitulate key aspects of cellular aging, each approach has potential limitations. Therefore, cross‐comparison between these orthogonal methods to identify reproducible and key attributes of cellular aging is crucial. Equally important is comparing these data in a different model (e.g., mouse models or human postmortem studies) to evaluate cellular aging in tissue, which we discuss further in this section. Noting that two of the fundamentally implicated cell types in ALS are motor neurons and astrocytes (recently reviewed in Serio & Patani, 2018), we specifically discuss benchmark studies of aging in these cell types.

## 6 MOTOR NEURONS

Motor neuron function progressively deteriorates with advancing age as the individual transitions from adulthood into old age. Aging is associated with slowing of gait, impaired balance, and deficits in fine motor skills, which cumulatively precipitate increased falls risk and carer dependence (Sorond et al., 2015). Although it is well established that with age, skeletal muscle, and neuromuscular junctions (NMJ) degenerate, the effect of age on motor neurons has only recently been elucidated from human postmortem tissue. Depletion of both upper and lower motor neurons and subsequent denervation of skeletal muscle contributes to sarcopenia and strength decline in elderly individuals (Rygiel, Grady, & Turnbull, 2014; Soreq et al., 2017). The reduction in motor neuron soma counts is followed by a decline in axonal density, with around 5% of axons lost every 10 years between the second and 10th decade of life (Kawamura, Okazaki, O'Brien, & Dych, 1977). By examining α motor neurons between species across a physiological age range, preservation of somal size was accompanied by the accumulation of lipofuscin deposits (cellular waste). Surprisingly, motor neurons did not atrophy with age; however, there was a significant reduction in cholinergic and glutaminergic synaptic inputs that directly abut motor neurons (Maxwell et al., 2018). Thus, it is the shedding of synaptic inputs, rather than motor neuron morphological changes that likely contribute to age‐related motor dysfunction.

One protein strongly implicated in motor neuron aging is the calcium‐dependent zinc containing endopeptidase, matrix metalloproteinase 1 (dMMP1). Using transcriptional profiling of a Drosophilia model, Azpura and coworkers not only reported that dMMP1 expression is age‐dependent, but that expression also correlates with a decline in motor function. Furthermore, by interrogating motor neuron transcriptomes across an age range, they found that overexpression of dMMP1 in young motor neurons mimics the effect of age on the motor system, with reduced NMJ neurotransmission and behavioral climbing deficits. Although MMP genes are crucial in the development of the nervous system, these findings indicate they elicit damaging consequences in later life (Azpurua, Mahoney, & Eaton, 2018). This combination of benefit in youth, but at the expense of harm post‐reproductive age, defines dMMP1 as an antagonistically pleiotropic gene.

Whether aging differentially affects subtypes of motor neurons is of great interest. Spinal motor neurons constitute a highly diverse population with various subclasses differing in gene expression and neuron function (William, Tanabe, & Jessell, 2003). Within motor neuron pools, there is a varied mix of slow‐twitch fatigue resistant, fast‐twitch fatigue resistant, and fast‐twitch fatigable α motor neurons. Interestingly, these subclasses display varying degrees of vulnerability to aging and degeneration in ALS, with fast‐twitch fatigable being the most vulnerable and slow‐twitch fatigue resistance being the most resistant (Chakkalakal, Nishimune, Ruas, Spiegelman, & Sanes, 2010). The mechanisms responsible for driving this differential susceptibility to motor neuron loss are likely to be multifactorial, with involvement of both intracellular influences (e.g., mitochondrial dysfunction, oxidative stress, calcium dyshomeostasis, and the accumulation of protein aggregates) and changes in extracellular signals (e.g., reduced IGF1 signaling and proinflammatory cytokines) (Ho et al., 2016).

## 7 ASTROCYTE AGING

A major component of the evolving “glial doctrine” is that astrocytes, the most abundant type of glial cell, trigger key molecular processes that lead to brain aging. Surprisingly, aging is characterized by a high percentage of astrocytes with a reactive phenotype, even when no sign of disease is apparent. Since mice lacking the microglial proinflammatory cytokines (IL‐1α, TNF and C1q) are protected against aging‐associated reactive astrogliosis, this suggests that microglia are at least in part responsible for inducing this *normal* brain “inflammaging” phenomenon (Clarke et al., 2018). The total astrocyte number within the CNS increases by 20% in the aged brain, whereas the number of oligodendrocytes and microglia remain constant (Pilegaard & Ladefoged, 1996). Aged astrocytes express higher levels of gap junctions and elevated levels of the cytoplasmic proteins, glial fibrillary acidic protein (GFAP), S100β (involved in calcium binding), and tau (although not neurofibrillary tangles) (Cotrina & Nedergaard, 2002).

Gene expression profiling from aging brains in mice has revealed that aging is associated with an inflammatory response and oxidative stress, with resemblance to human neurodegeneration (Lee, Weindruch, & Prolla, 2000). The GFAP gene undergoes a twofold increase in gene expression, which maybe in response to these injurious stimuli. Indeed, inflammation and oxidative stress have been shown to induce alterations in astrocyte calcium signaling, thereby impacting on astrocytic modulation of neuronal function (Squier & Bigelow, 2000). Using RNA sequencing of astrocytes from different brain regions across the lifespan of the mouse, Clarke and coworkers were able to demonstrate that aged astrocytes significantly upregulate several potentially detrimental astrocyte reactive genes. Distinguishing astrocyte changes between *normal* brain aging and early *pathological* neurodegeneration will be a crucial challenge in order to identify specific therapeutic targets.

## 8 SUMMARY

Over the past decade, the iPSC field has made significant advances permitting generation of unlimited numbers of any differentiated cell type with the genetics of diseased patients. Producing these models has provided a platform to investigate cellular mechanisms of the disease, thus releasing the brake on the understanding of human neurological diseases, which have been notoriously challenging to study due to difficulties accessing patient tissue. Although differentiation protocols provide a novel perspective that cannot be achieved from animal models, differentiating rejuvenated cells from patients with ALS has thus far not fully integrated cellular aging into disease modeling paradigms. Across the iPSC spectrum, differentiated cells repeatedly retain embryonic characteristics and lack the crucial aging component that precedes the disease.

The issue facing the field now is whether we can complement the iPSC platform with an induced aging paradigm that incorporates aging‐associated molecular pathology. To overcome this hurdle, numerous strategies have been employed to experimentally manipulate aging in vitro; nonetheless, the field still requires a systematic and comprehensive multi‐pronged approach that addresses the different cellular and molecular hallmarks of aging in a standardized and reproducible manner. Recent work replicating complex in vivo conditions has improved disease models but whether this helps to recapture aging per se remains to be determined. Although these approaches hasten the development of disease‐related phenotypes, given their artificial nature, it must be questioned whether they authentically recapitulate the aging process. The capacity to reprogram and differentiate cells that are fully functional and appropriately aged bestows a vital milestone on the path to generating optimal iPSC models of ALS.

## CONFLICTS OF INTEREST

O.J.Z. and R.P. have no competing interests.

## AUTHORS CONTRIBUTION

O.J.Z. produced the original draft of the manuscript with conceptual guidance from R.P., who revised the manuscript. O.J.Z. and R.P. finalized the work.
