# Supplementary material for: Harnessing cellular aging in human stem cell models of amyotrophic lateral sclerosis
Source: Aging Cell. 2018 Dec 19;18(1):e12862. doi: 10.1111/acel.12862 (PMC6351881; doi:10.1111/acel.12862)
Supplement: Supplementary file 1 [file ACEL-18-e12862-s001.docx]

## Supplementary Table. Information Box S1: *In vitro* culture considerations to model disease

1. **Prolonged Culture**

A straightforward and physiologically representative approach to mimic aging is to extend the culture for several months, providing time for the intrinsic clock to accrue damage. Comparing 8 week vs. 15 week culture demonstrated that prolonged differentiation enabled the crucial developmental switch from neurogenesis to astrogliogenesis and enhances functional maturation of iPSC derived neuronal cultures. However, prolonged culture has significant challenges including large variability in the propensity for endogenous astrogliogenesis and functional maturation (Paavilainen et al., 2018). These basal culture conditions often prove experimentally laborious and insufficient in invoking other aspects of the ALS phenotype.

1. **Genome editing using CRISPR/Cas9**

The direct manipulation of the genome using editing tools may greatly facilitate efforts to model aging through the systematic targeting of identified aging pathways. This approach was utilised in short-lived African killifish, where multiple genes encompassing the hallmarks of aging were mutated. As a proof of principle, the authors successfully demonstrated the genome-to-phenotype platform by exposing telomere related pathologies in fish with telomerase mutations (Harel et al., 2015). This unique approach may enable the high throughput investigation of candidate aging genes arising from human genome wide studies.

1. **Toxic stress**

Cellular stressors including reactive oxygen species (ROS), inducers of mitochondrial dysfunction and poisons such as arsenic and pesticides such as rotenone have been utilised to elicit an aging phenotype in neural tissue (Garcia, Fernandez, & Solas, 2013; Nishitoh et al., 2008; Sim, Zhao, Penderis, & Franklin, 2002). These stressors reduce the time necessary for accruing cellular injury that ensues throughout a complete lifetime (Lin & Beal, 2006; Sanchez-Danes et al., 2012). In ALS, metabolic changes within mitochondria of motor neurons activate pro-apoptotic pathways via abnormal calcium fluxes and enhanced sensitivity to ROS (Estevez et al., 1999; Garcia et al., 2013; Manfredi & Xu, 2005). Furthermore, oxidative stress with PI3K inhibitors precipitates TDP43 aggregate formation and motor neuron death (Bilican et al., 2012; Burkhardt et al., 2013; Egawa et al., 2012). Nonetheless, selected targeting of these stress-pathways risks evading other features that may also be involved in aging and ALS pathogenesis.

1. **Co-cultures of neurons and astrocytes**

The interactions between neurons and astrocytes leads to growth promoting effects on the CNS with astrocytes in co-culture stimulating the functional maturation of the neighbouring iPSC derived neurons. Combining differentiating neurons with astrocytes significantly improved both the morphological and functional development of neurons, when compared to neurons cultured alone (Pfrieger & Barres, 1997). Astrocytes induced differentiation efficiency, dendritic branching and maturation in electrophysiology and synaptic properties in adjacent neurons (Tang et al., 2013). Studying non-cell autonomous influences on neuronal maturation has been attempted with motor neurons cultured with skeletal muscle to recreate the NMJ and revealed the importance of recapitulating cellular interactions in guiding cell maturation (Das, Rumsey, Bhargava, Stancescu, & Hickman, 2010). These findings emphasise the significance of restoring the physiological conditions for cell fate determination to accurately model disease *in vitro*.

1. **Organoids**

To reproduce the in vivo architecture of a tissue, three dimensional (3D) culture techniques of iPSCs have been utilised to produce “organoids.” This has been achieved with artificial scaffolds allowing cells to form very organised structures (Ader & Tanaka, 2014). Cerebral and retinal organoids have successfully been produced yielding high fidelity multi-layered structures with resembling morphology and neuronal subtype composition (Eiraku et al., 2011; Eiraku et al., 2008; Lancaster et al., 2013). Using polymeric scaffolds, cortical neurons have been produced in a layered organisation, which improved neuronal functional connectivity.(Kunze, Giugliano, Valero, & Renaud, 2011) Furthermore, using a 3D iPSC model of Alzheimer’s disease, overexpressing mutant β amyloid precursor protein and presenilin 1 into matrigel droplets, successfully produced for the first time β amyloid plaques and neurofibrillary tangles *in vitro*.(Choi et al., 2014) Combining the cerebral organoid approach with prolonged culture (up to 6 months) led to inhibitory and excitatory neurons together with oligodendrocytes which took on a more mature phenotype compared with cerebral organoids alone, which undergo short cultivation periods (Matsui et al., 2018).

1. **Microfluidics**

Microfluidic systems are microscale devices designed to precisely regulate fluid exchanges. This organ on a chip method has been utilised to more accurately simulate the physiological conditions of the CNS, by fine-tuning nutrient and growth factor concentrations to support cell development. This approach has successfully generated key cerebral structures including the BBB (Griep et al., 2013) and neurovascular units (Achyuta et al., 2013). To date only a handful of studies have incorporated these technologies with iPSC differentiation protocols, likely as a result of both the technical difficulties and costs of recapitulating the organ microenvironment. Nonetheless, increasing the intricacy of the *in vitro* environment holds abundant potential to impersonate human tissue.

1. **Bioengineered substrates**

Mechanical platforms that replicate cell-matrix interactions to culture iPSCs enhance motor neuron differentiation. Using advances in micromolded poly-dimethylsiloxane micropost arrays (PMA), iPSCs have been successfully cultured into motor neurons on vitronectin-coated soft PMA surface with a >4-fold increase in differentiation efficiency, thus significantly reducing the lag required for maturation (Fu et al., 2010). PMAs have a constant surface geometry whose rigidity varies with altering heights of microposts, replicating the stiffness range within soft tissues whilst allowing tethering of cell-surface integrins. Varying the substrate rigidity has been shown to influence mechanotransduction and cell fate during iPSC differentiation, suggesting that tension-dependent mechanotransduction is important for regulating stem cell differentiation (Sun et al., 2014). Efficient *in vitro* derivation of large-scale motor neurons from iPSCs was achieved using an optimised extracellular matrix of small molecules combined with seeding cells on a cocktail of four matrix proteins (Qu et al., 2014). This method produced rapid, highly efficient and high-yield production of mature and functional motor neurons from human iPSCs. Interestingly, neurite branching is increased on smooth microposts but reduced on microgratings and DNA microarray analysis identified 20 differentially expressed genes expressed in neurons reprogrammed on smooth versus rough surfaces (Kulangara et al., 2014).

Achyuta, A. K., Conway, A. J., Crouse, R. B., Bannister, E. C., Lee, R. N., Katnik, C. P., . . . Sundaram, S. S. (2013). A modular approach to create a neurovascular unit-on-a-chip. *Lab Chip, 13*(4), 542-553. doi:10.1039/c2lc41033h

Ader, M., & Tanaka, E. M. (2014). Modeling human development in 3D culture. *Curr Opin Cell Biol, 31*, 23-28. doi:10.1016/j.ceb.2014.06.013

Bilican, B., Serio, A., Barmada, S. J., Nishimura, A. L., Sullivan, G. J., Carrasco, M., . . . Chandran, S. (2012). Mutant induced pluripotent stem cell lines recapitulate aspects of TDP-43 proteinopathies and reveal cell-specific vulnerability. *Proc Natl Acad Sci U S A, 109*(15), 5803-5808. doi:10.1073/pnas.1202922109

Burkhardt, M. F., Martinez, F. J., Wright, S., Ramos, C., Volfson, D., Mason, M., . . . Javaherian, A. (2013). A cellular model for sporadic ALS using patient-derived induced pluripotent stem cells. *Mol Cell Neurosci, 56*, 355-364. doi:10.1016/j.mcn.2013.07.007

Choi, S. H., Kim, Y. H., Hebisch, M., Sliwinski, C., Lee, S., D'Avanzo, C., . . . Kim, D. Y. (2014). A three-dimensional human neural cell culture model of Alzheimer's disease. *Nature, 515*(7526), 274-278. doi:10.1038/nature13800

Das, M., Rumsey, J. W., Bhargava, N., Stancescu, M., & Hickman, J. J. (2010). A defined long-term in vitro tissue engineered model of neuromuscular junctions. *Biomaterials, 31*(18), 4880-4888. doi:10.1016/j.biomaterials.2010.02.055

Egawa, N., Kitaoka, S., Tsukita, K., Naitoh, M., Takahashi, K., Yamamoto, T., . . . Inoue, H. (2012). Drug screening for ALS using patient-specific induced pluripotent stem cells. *Sci Transl Med, 4*(145), 145ra104. doi:10.1126/scitranslmed.3004052

Eiraku, M., Takata, N., Ishibashi, H., Kawada, M., Sakakura, E., Okuda, S., . . . Sasai, Y. (2011). Self-organizing optic-cup morphogenesis in three-dimensional culture. *Nature, 472*(7341), 51-56. doi:10.1038/nature09941

Eiraku, M., Watanabe, K., Matsuo-Takasaki, M., Kawada, M., Yonemura, S., Matsumura, M., . . . Sasai, Y. (2008). Self-organized formation of polarized cortical tissues from ESCs and its active manipulation by extrinsic signals. *Cell Stem Cell, 3*(5), 519-532. doi:10.1016/j.stem.2008.09.002

Estevez, A. G., Crow, J. P., Sampson, J. B., Reiter, C., Zhuang, Y., Richardson, G. J., . . . Beckman, J. S. (1999). Induction of nitric oxide-dependent apoptosis in motor neurons by zinc-deficient superoxide dismutase. *Science, 286*(5449), 2498-2500.

Fu, J., Wang, Y. K., Yang, M. T., Desai, R. A., Yu, X., Liu, Z., & Chen, C. S. (2010). Mechanical regulation of cell function with geometrically modulated elastomeric substrates. *Nat Methods, 7*(9), 733-736. doi:10.1038/nmeth.1487

Garcia, M. L., Fernandez, A., & Solas, M. T. (2013). Mitochondria, motor neurons and aging. *J Neurol Sci, 330*(1-2), 18-26. doi:10.1016/j.jns.2013.03.019

Griep, L. M., Wolbers, F., de Wagenaar, B., ter Braak, P. M., Weksler, B. B., Romero, I. A., . . . van den Berg, A. (2013). BBB on chip: microfluidic platform to mechanically and biochemically modulate blood-brain barrier function. *Biomed Microdevices, 15*(1), 145-150. doi:10.1007/s10544-012-9699-7

Harel, I., Benayoun, B. A., Machado, B., Singh, P. P., Hu, C. K., Pech, M. F., . . . Brunet, A. (2015). A platform for rapid exploration of aging and diseases in a naturally short-lived vertebrate. *Cell, 160*(5), 1013-1026. doi:10.1016/j.cell.2015.01.038

Kulangara, K., Adler, A. F., Wang, H., Chellappan, M., Hammett, E., Yasuda, R., & Leong, K. W. (2014). The effect of substrate topography on direct reprogramming of fibroblasts to induced neurons. *Biomaterials, 35*(20), 5327-5336. doi:10.1016/j.biomaterials.2014.03.034

Kunze, A., Giugliano, M., Valero, A., & Renaud, P. (2011). Micropatterning neural cell cultures in 3D with a multi-layered scaffold. *Biomaterials, 32*(8), 2088-2098. doi:10.1016/j.biomaterials.2010.11.047

Lancaster, M. A., Renner, M., Martin, C. A., Wenzel, D., Bicknell, L. S., Hurles, M. E., . . . Knoblich, J. A. (2013). Cerebral organoids model human brain development and microcephaly. *Nature, 501*(7467), 373-379. doi:10.1038/nature12517

Lin, M. T., & Beal, M. F. (2006). Mitochondrial dysfunction and oxidative stress in neurodegenerative diseases. *Nature, 443*(7113), 787-795. doi:10.1038/nature05292

Manfredi, G., & Xu, Z. (2005). Mitochondrial dysfunction and its role in motor neuron degeneration in ALS. *Mitochondrion, 5*(2), 77-87. doi:10.1016/j.mito.2005.01.002

Matsui, T. K., Matsubayashi, M., Sakaguchi, Y. M., Hayashi, R. K., Zheng, C., Sugie, K., . . . Mori, E. (2018). Six-month cultured cerebral organoids from human ES cells contain matured neural cells. *Neurosci Lett, 670*, 75-82. doi:10.1016/j.neulet.2018.01.040

Nishitoh, H., Kadowaki, H., Nagai, A., Maruyama, T., Yokota, T., Fukutomi, H., . . . Ichijo, H. (2008). ALS-linked mutant SOD1 induces ER stress- and ASK1-dependent motor neuron death by targeting Derlin-1. *Genes Dev, 22*(11), 1451-1464. doi:10.1101/gad.1640108

Paavilainen, T., Pelkonen, A., Makinen, M. E., Peltola, M., Huhtala, H., Fayuk, D., & Narkilahti, S. (2018). Effect of prolonged differentiation on functional maturation of human pluripotent stem cell-derived neuronal cultures. *Stem Cell Res, 27*, 151-161. doi:10.1016/j.scr.2018.01.018

Pfrieger, F. W., & Barres, B. A. (1997). Synaptic efficacy enhanced by glial cells in vitro. *Science, 277*(5332), 1684-1687.

Qu, Q., Li, D., Louis, K. R., Li, X., Yang, H., Sun, Q., . . . Wang, F. (2014). High-efficiency motor neuron differentiation from human pluripotent stem cells and the function of Islet-1. *Nat Commun, 5*, 3449. doi:10.1038/ncomms4449

Sanchez-Danes, A., Richaud-Patin, Y., Carballo-Carbajal, I., Jimenez-Delgado, S., Caig, C., Mora, S., . . . Raya, A. (2012). Disease-specific phenotypes in dopamine neurons from human iPS-based models of genetic and sporadic Parkinson's disease. *EMBO Mol Med, 4*(5), 380-395. doi:10.1002/emmm.201200215

Sim, F. J., Zhao, C., Penderis, J., & Franklin, R. J. (2002). The age-related decrease in CNS remyelination efficiency is attributable to an impairment of both oligodendrocyte progenitor recruitment and differentiation. *J Neurosci, 22*(7), 2451-2459. doi:20026217

Sun, Y., Yong, K. M., Villa-Diaz, L. G., Zhang, X., Chen, W., Philson, R., . . . Fu, J. (2014). Hippo/YAP-mediated rigidity-dependent motor neuron differentiation of human pluripotent stem cells. *Nat Mater, 13*(6), 599-604. doi:10.1038/nmat3945

Tang, X., Zhou, L., Wagner, A. M., Marchetto, M. C., Muotri, A. R., Gage, F. H., & Chen, G. (2013). Astroglial cells regulate the developmental timeline of human neurons differentiated from induced pluripotent stem cells. *Stem Cell Res, 11*(2), 743-757. doi:10.1016/j.scr.2013.05.002
